# Intergenerational transmission of child maltreatment using a multi-informant multi-generation family design

**DOI:** 10.1371/journal.pone.0225839

**Published:** 2020-03-12

**Authors:** Renate S. M. Buisman, Katharina Pittner, Marieke S. Tollenaar, Jolanda Lindenberg, Lisa J. M. van den Berg, Laura H. C. G. Compier-de Block, Joost R. van Ginkel, Lenneke R. A. Alink, Marian J. Bakermans-Kranenburg, Bernet M. Elzinga, Marinus H. van IJzendoorn

**Affiliations:** 1 Centre for Forensic Family and Youth Care Studies, Leiden University, Leiden, The Netherlands; 2 Leiden Institute for Brain and Cognition (LIBC), Leiden University, Leiden, Netherlands; 3 Institute of Psychology, Clinical Psychology Unit, Leiden University, Leiden, The Netherlands; 4 Leyden Academy on Vitality and Ageing, Leiden, The Netherlands; 5 Methodology and Statistics, Institute of Psychology, Leiden University, Leiden, The Netherlands; 6 Faculty of Behavioural and Movement Sciences, Vrije Universiteit Amsterdam, Amsterdam, The Netherlands; 7 Primary Care Unit, School of Clinical Medicine, University of Cambridge, Cambridge, United Kingdom; 8 Department of Psychology, Education and Child Studies, Erasmus University Rotterdam, Rotterdam, The Netherlands; Monash University, AUSTRALIA

## Abstract

In the current study a three-generational design was used to investigate intergenerational transmission of child maltreatment (ITCM) using multiple sources of information on child maltreatment: mothers, fathers and children. A total of 395 individuals from 63 families reported on maltreatment. Principal Component Analysis (PCA) was used to combine data from mother, father and child about maltreatment that the child had experienced. This established components reflecting the convergent as well as the unique reports of father, mother and child on the occurrence of maltreatment. Next, we tested ITCM using the multi-informant approach and compared the results to those of two more common approaches: ITCM based on one reporter and ITCM based on different reporters from each generation. Results of our multi-informant approach showed that a component reflecting convergence between mother, father, and child reports explained most of the variance in experienced maltreatment. For abuse, intergenerational transmission was consistently found across approaches. In contrast, intergenerational transmission of neglect was only found using the perspective of a single reporter, indicating that transmission of neglect might be driven by reporter effects. In conclusion, the present results suggest that including multiple informants may be necessary to obtain more valid estimates of ITCM.

## Introduction

What puts parents at risk to maltreat their children? This is a question that has been the subject of research for several decades [1,2]. One prevailing hypothesis is that child maltreatment is passed down through family trees, moving from one generation to the next. This notion has been approached from multiple, albeit different, theoretical perspectives, including social-learning [3], developmental psychopathology [4], and attachment theory [5]. However, the empirical evidence for intergenerational transmission of child maltreatment (ITCM) has been mixed. Although ITCM was found in some studies [6–8], other researchers have found no evidence for transmission [1,9,10]. Overall, meta-analytic evidence suggests that there is ITCM but that effect sizes are modest [11].

These mixed results can be partly attributed to variations in design (e.g., retrospective vs. prospective), population, and sampling strategy (e.g., at risk vs. representative sample), and source of maltreatment reports (e.g., official records vs. child or parent report). One methodological aspect of ITCM which has not received much attention is the use of single informant vs. multi-informant approaches. Multi-informant approaches offer advantages over single informant approaches such as reducing error and individual bias [12]. In the current study we apply a multi-generational family design to compare ITCM using single informant approaches to a multi-informant approach.

**Fig 1:**
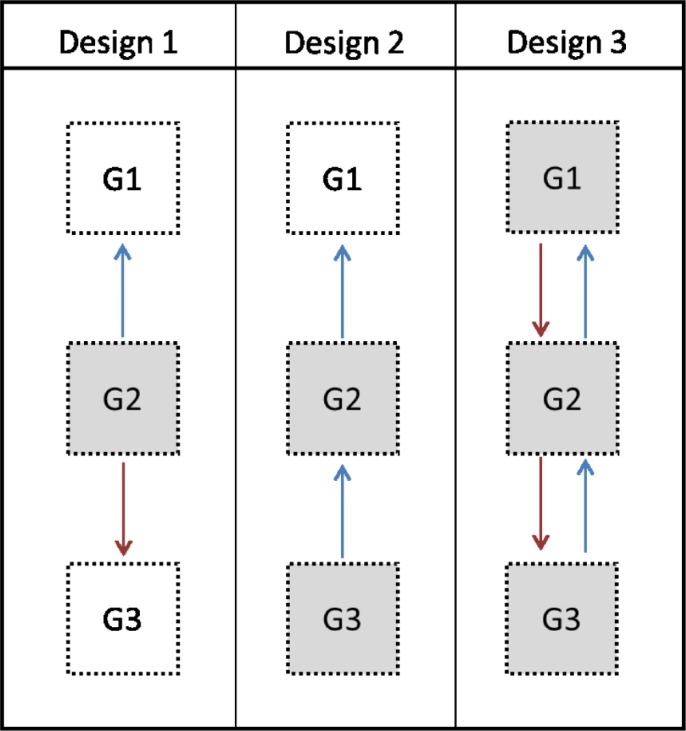
Three designs to test intergenerational transmission of child maltreatment (ITCM) were used. (1) One informant reports on experienced and perpetrated maltreatment (G2, in grey), (2) Informants from two generations (G2 and G3) report about experienced maltreatment, and (3) Informants from three generations report about experienced and perpetrated maltreatment (G1, G2, and G3). G1 = Generation 1; G2 = Generation 2; G3 = Generation 3; Blue arrow = child report; Red arrow = parent report.

A multi-generation family design offers opportunities to address specific ITCM issues. So far, few studies have employed this kind of design, possibly because such studies are methodologically complex and recruiting families, compared to individual participants, is clearly more challenging. Using a one-generational design, ITCM can be tested by asking the participants (G2) about any maltreatment they have experienced and any maltreatment they have perpetrated (see Fig 1, Design 1). However, this approach is vulnerable to overestimation of transmission if a common factor, e.g., recall bias, affects both the report on experienced maltreatment and the report on perpetrated maltreatment. One way to prevent this is to use a two-generational design by including a second generation. In that case, participants from both generations report whether they have experienced childhood maltreatment. This design is stronger but it cannot be extended to include multiple reporters of maltreatment experienced by the parent (G2, see Fig 1, Design 2). By extending this design vertically (by including additional generations) and horizontally (multiple siblings, nephews, nieces etc.), estimates can be based on multiple reporters, as it is possible to include reports from both parents and multiple children both about experienced and perpetrated maltreatment. Reporter bias can thus be reduced by including three participating generations (Fig 1, Design 3). Moreover, several siblings can report on the maltreatment perpetrated by the same parent giving a more comprehensive picture of parents’ behavior. The 3G Parenting Study utilized a multi-informant multigenerational cross-sectional extended family study design (Supplementary Material S1 Text). The aim of the current paper is to empirically test ITCM using this design while also addressing reporter effects.

### Intergenerational transmission using multiple informants

Studies on ITCM have almost exclusively used self-report to measure experienced and perpetrated maltreatment in parents, with a few exceptions that used official reports [13]. The use of official reports (i.e., Child Protection Services (CPS) reports) may result in a more “objective” rating of a sensitive issue than using parental or child self-report of child maltreatment, since it relies on observations of professionals. A major disadvantage of this approach, however, is that maltreatment may go unnoticed by professionals and only the most severe cases are usually substantiated by CPS [14]. Parental and child self-report of child maltreatment are more likely to capture the whole range of maltreatment experiences. Parents may however underreport perpetrated maltreatment because of social stigma and legal consequences [15]. Conversely, children may underreport experienced maltreatment for various reasons, including loyalty to their parents or fear of punishment [16,17]. In addition, both parent and child reports may be biased due to distorted memories [18], or due to their subjective appraisals of certain experiences, or lack of experiences, as in the case of neglect [19]. These biases can be alleviated by including different perspectives. This however creates new challenges, particularly if these perspectives differ. In most cases, there are no theoretical or empirical reasons to prioritize one reporter over the other and there is no singularly right way to combine several reports.

Convergence between parent-reported and child-reported incidence of maltreatment is generally low to moderate [20–22]. Nonetheless, precisely this divergence makes it important to include several reporters, since different reports may lead to different conclusions [23] and because the meaning of differences between reporters is not well understood [24]. A better understanding of reporter effects also has important practical implications. Professionals involved in decision making about interventions in case of maltreatment (including out-of-home placements) often take into account the reports of parent and children in their decision making [25]. Understanding the implications of parent-child convergence and divergence may help professionals to make better decisions.

Reporter effects may play a considerable role in ITCM. For instance, a prospective cohort study examined ITCM in a sample of maltreatment victims confirmed by Child Protective Services (CPS) and a matched comparison group [26]. Parent report, child report and reports from CPS as measures of perpetrated maltreatment were compared. A complex pattern of ITCM emerged indicating that transmission depended on reporter and type of maltreatment. Support for the transmission of neglect was found irrespective of the reporter. There was transmission of sexual abuse when perpetrated maltreatment was measured using CPS records and child report but not parent report. However, transmission of physical abuse was only detected using CPS records. The authors concluded that “the extent of the intergenerational transmission of abuse and neglect depend[s] in large part on the source of the information used to assess maltreatment” [26] To further understand and interpret this finding, in the current study we included multiple sources of information on (experienced and perpetrated) abuse and neglect, i.e., mothers, fathers and children, and tested various models to examine ITCM.

### An alternative approach of integrating different reports

Currently no gold standard exists on how to combine information from different informants. It has been proposed to average the scores of different informants [27,28]. However, this approach does not enhance our understanding of differences between reporters. Others have argued that scores of different informants can best be analyzed separately when the level of agreement between informants is low [29,30]. A limitation of this strategy is that results obtained from models with different informant scores may be inconsistent and challenging to interpret. Findings are tied to a specific informant and therefore difficult to generalize. Other approaches–which are common in psychiatric research but may also be used in child maltreatment research–are to combine information from each source with the “OR” rule, classifying the condition as present when reported by at least one informant, or the “AND” rule, classifying the condition as present when reported by all informants [12]. The disadvantage of both approaches, however, is that valuable information could be overlooked, since more often than not parents and children have different perspectives which may both be valid to some extent [31,32]. Moreover, these approaches rely on dichotomies, whereas continuous scores may provide important information on the variance in extent, severity, and chronicity of child maltreatment.

The aim of the present study was to integrate reports from multiple informants to test ITCM. The method used was devised by Kraemer et al. [12] to deal with inter-informant disagreement in psychiatric assessments. Traditionally, disagreement is viewed as noise which should be eliminated. Alternatively, this disagreement, in part, reflects the unique perspectives of each reporter–information the other reporters do not have access to but is part of a complete assessment. Therefore, Kraemer et al. [12] argued for an integrative method in which the shared (i.e., convergent) perspective, and the unique (i.e., discordant) perspectives of each informant should be extracted using Principal Component Analysis (PCA). Sierau et al. [33] applied this approach to the measurement of child maltreatment, and established three components in their study: (1) the shared perspective between parent, child and CPS on the occurrence of maltreatment (convergence), (2) the child’s unique perspective, and (3) the parent versus CPS perspective.

Applied to our study, we aimed to establish components reflecting the shared (convergent) as well as the unique (discordant) perspectives of father, mother, and child on the occurrence of maltreatment. Child maltreatment was measured on a continuous scale with a range from no child maltreatment, over milder forms of child maltreatment, to severe child maltreatment. In clinical and legal contexts, child maltreatment is often assessed binary (i.e., absent/present), but this cutoff is rather arbitrary for research purposes. Moreover, using a continuous measure of maltreatment is in accordance with current developments toward continuous models of psychopathology [34]. We employed Kraemer et al.’s (2003) data-driven approach to integrate data on maltreatment from father, mother, and child, allowing for unknown or unexpected patterns of inter-informant concordance and discordance. Subsequently, ITCM was tested by using the extracted convergence and discordance components as predictors of perpetrated maltreatment. To compare the results of this approach to more conventional approaches, ITCM was also estimated using the perspective of one reporter and the perspective of different reporters from each generation:

1. Based on theoretical and empirical evidence, we expect that ITCM will be found for maltreatment when using a one-generational design (Fig 1, Design 1), i.e., one informant reports about both experienced and perpetrated maltreatment.
2. We expect ITCM when informants from two generations report on experienced and perpetrated maltreatment (Fig 1, Design 2) based on theory but empirical evidence is mixed.
3. We expect ITCM when informants from three generations report on experienced and perpetrated maltreatment (Fig 1, Design 3) based on theory but empirical evidence is lacking.

Additionally, we explored the role of divergent reports in ITCM. Lastly, different patterns of ITCM for abuse and neglect were explored. It has been argued that experiences of threat, such as abuse, and experiences of deprivation, such as neglect, affect development differently [35], but it is unclear what implications this has for the intergenerational transmission. In all analyses we, therefore, distinguished between abuse and neglect.

## Method

### Recruitment

In order to increase power to detect intergenerational transmission of child maltreatment, we oversampled for experienced maltreatment by recruiting target participants from three participant pools: (1) The Netherlands Study of Depression and Anxiety (NESDA [36]), (2) the Longitudinal Internet Studies for the Social Sciences (LISS panel [37]) and (3) a study on parenting [38]. From two of these studies, maltreatment information was available and only participants with a known history of maltreatment were asked to participate in the 3G Parenting Study. From the third study, all participants were invited. In order to protect the privacy of the participants we cannot disclose from which study we recruited maltreated participants. Participants were sent a flyer about the study, and were subsequently contacted by phone. When participants agreed to take part in the study, we asked permission to invite their partners and family members (parents, children, siblings (and their partners), nieces, and nephews) to participate as well. Family members had to be at least 7.5 years of age to be included. Families were included if at least two first-degree relatives from two generations agreed to participate. Participants were informed about the general aim of the study (which was formulated as the role of genes and parenting in the intergenerational transmission of stress-related traits) and about the procedure of a lab visit.

### Sample

**Fig 2:**
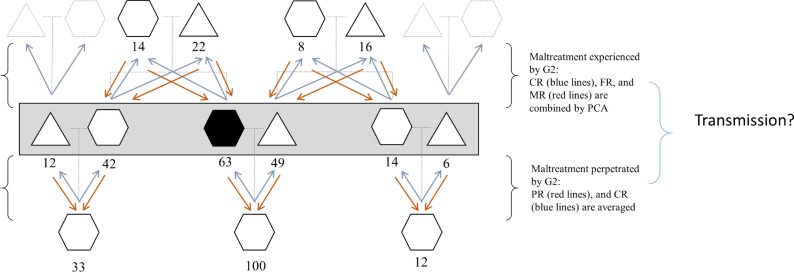
Summary family tree of participants. Black hexagon = Target participant (recruited first); Dotted symbols: Children reported about these parents but parents were never invited to the lab; Red arrow = Reports of perpetrated maltreatment. Blue arrow = Reports of experienced maltreatment. CR = Child report; FR = Father report; MR = mother report; PR = Parent report. Five participants were omitted from this diagram for simplicity: One partner from the third generation (G3) and three participants from the fourth generation.

In the 3G Parenting Study 63 families of 395 individuals from up to four generations participated (Fig 2), with an average of 6.27 family members per family (range: 2 to 23). There were 32 families comprising of two generations, 30 families comprising of three generations, and one family comprising of four generations. Generations were defined on the basis of the target participant (first recruited). This participant was always assigned to the second generation (G2). The first generation (G1) consisted of 60 participants (63% female, *M*_age_ = 68.92 years, range_age_ = 53.25 to 88.42 years) and reported about maltreatment experienced at the hands of their parents (“G0”, father and mother separately) and about maltreatment perpetrated towards the second generation (G2). In the second generation (G2) 186 participants were included (57% female, *M*_age_ = 45.98 years, range_age_ = 21.17 to 69.67 years) and reported about maltreatment experienced at the hands of their parents (G1, father and mother separately) and about maltreatment perpetrated towards their children (G3). The third generation (G3; *n* = 146, 55% female; M_age_ = 17.97 years, range_age_ = 7.50 to 47.50 years) reported about maltreatment experienced at the hands of their parents (G2, father and mother separately). In a minority of cases G3 had children and reported about perpetrated maltreatment towards their children (G4; *n* = 16). Three G4 participants were included in the current study and reported about maltreatment perpetrated by G3 (father and mother separately). We used Analysis of Covariance (ANCOVA) to compare participating fathers (G1 and G2, *n* = 164) to fathers who were eligible but did not participate (*n* = 78) on perpetrated abuse (child report). We controlled for child age and gender. Based on child report, participating fathers perpetrated more abuse than fathers who did not participate (*F*(1, 238) = 7.67, *p* < .01). Conversely, fathers who did not participate were reported to be more neglecting (*F*(1, 238) = 25.53, *p* < .001). The same pattern was found for participating (*n* = 202) and non-participating mothers (*n* = 55; abuse: *F*(1, 253) = 5.69, *p* = .02; neglect: *F*(1, 253) = 4.30, *p* = .04).

Of the adult participants (≥ 18 years, *n* = 302) 6% completed elementary school, 19% lower vocational school, 40% completed advanced secondary education, and 28% had a college or university degree (6% unknown). The sample was rather homogenous in terms of ethnicity: 96% of the participants were Caucasian.

### Procedure

Participants and their families were invited to the lab for one or two days, depending on family composition. Participants from the second and third generation with children visited the lab once with their nuclear family and once with their family of origin. In some cases family members attended the lab sessions separately for practical or personal reasons. During the lab visits, participants individually completed questionnaires and computer tasks, and did several interaction tasks together with their family members. During some of the tasks heart rate and skin conductance were measured [39]. To measure hormone levels and DNA saliva, hair, and buccal samples were collected. Eligible participants were also invited for a functional magnetic resonance imaging (fMRI) session [40]. Questionnaires on child maltreatment were completed during the first visit. Since all participants with children completed at least two of these questionnaires (one on experienced and one on perpetrated maltreatment), these questionnaires were scheduled as far apart as possible. The study was approved by the ethics committee of the Leiden University Medical Centre (reference number: P11.134). Written informed consent was obtained from all participants before participation. For participants under 18 years of age, parents cosigned informed consent. As a compensation for participation, adults received 50 Euros for one lab visit and up to 100 Euros (depending on time investment) for two lab visits, as well as travelling expenses. Data was collected between March 2013 and May 2016.

### Ethical considerations

In the Netherlands, the Protocol Reporting Code Domestic Violence and Child Abuse applies. This means that if a child under 18 years of age reports maltreatment, individuals working in health care, child or youth care, education, social support, and criminal justice are obligated to make a report. After every lab visit with children under 18 years, one of the first authors of this paper checked the parent and child reported maltreatment questionnaires. Relevant cases were (anonymously) reviewed by the research team (including senior researchers with clinical experience). In cases where current moderate to severe child maltreatment was suspected a senior psychologist discussed the case with a clinical psychologist of a specialized center for psychological trauma. If the family was not under legal or clinical guidance already, the local Advice and Reporting Centre for Child Abuse and Neglect (*Veilig Thuis*) was subsequently contacted. In accordance with the recommendation from this Centre one of the following steps were taken: (1) no action, (2) the family was contacted to gain further information, or (3) a report was filed and appropriate action was taken by the Advice and Reporting Centre for Child Abuse and Neglect. Ultimately, the Advice and Reporting Centre for Child Abuse and Neglect was contacted about three families.

### MeasuresChild maltreatment

Experienced and perpetrated child maltreatment were measured using a combination of the Parent-Child Conflict Tactics Scales (CTS-PC: [41] and the Childhood Trauma Questionnaire (CTQ: [42,43]). The CTS-PC originally consists of four scales. However, we excluded the *Nonviolent Discipline* scale (4 items), because it does not include items on maltreatment. The *Psychological Aggression* scale (i.e., emotional abuse), consisting of 5 items, assesses verbal or other nonphysical communication aimed at inflicting psychological pain or fear to the child (e.g., “Shouted, yelled, or screamed”). *Physical Assault* (i.e., physical abuse) is comprised of 13 items, including corporal punishment (5 items, e.g., “Spanked on the bottom with a bare hand”), severe assault (4 items, e.g., “Hit with a fist or kicked hard”), and very severe assault (4 items, e.g., “Burned or scalded”). The *Neglect* scale consists of 5 items and measures the failure of a parent to engage in behavior that is necessary to meet the developmental needs of a child (e.g., “My father/mother was not able to make sure I got the food I needed”). Since the *Neglect* scale includes only one item on emotional neglect (“My father/mother never told me he/she loved me”), we added five items of the *Emotional Neglect* scale of the CTQ [42] reverse coded for the purpose of analysis. To match the response categories of the CTS and CTQ, we used a 5-point scale ranging from 1 = *never* to 5 = *almost always* for all items.

Participants completed a version that assessed the extent to which they had experienced specific physically or psychologically neglectful or aggressive behaviors from their father and/or mother before the age of 18 years. Participants with children reported the extent to which they had conducted these behaviors towards (each of) their child(ren). For participants under 12 years of age, experienced maltreatment was assessed orally and questions about very severe physical abuse were omitted. Participants aged 12–18 years and living with their parents at the time of the study indicated whether they had experienced maltreatment within the last year or in the years before. Per item, the higher score of these two was included in all calculations. Subscale scores based on the higher score correlated significantly with subscales based on either last year (range: r(47) = .40 –.88) or the years before (range: r(46) = .86 –.99). For participants aged 18 years or older, lifetime maltreatment (until 18 years) was assessed.

Internal consistencies of the scales for experienced maltreatment were as follows: for physical abuse α_mother_ = .92 and α_father_ = .92, for emotional abuse α_mother_ = .78 and α_father_ = .73, for physical neglect α_mother_ = .65 and α_father_ = .57 and for emotional neglect α_mother_ = .91 and α_father_ = .89. Internal consistencies of the scales on perpetrated maltreatment were: for physical abuse α_child1_ = .71 and α_child2_ = .76, for emotional abuse α_child1_ = .69 and α_child2_ = .66, for physical neglect α_child1_ = .38 and α_child2_ = .36 for, and for emotional neglect α_child1_ = .69 and α_child2_ = .67. We initially aimed to distinguish these four types of maltreatment. However, internal consistencies for the four items on physical neglect were not sufficient and the physical abuse and the physical neglect scale were both highly skewed to the right. We therefore decided to combine the physical and emotional scales. Internal consistencies of these combined scales were as follows: for experienced abuse α_mother_ = .92 and α_father_ = .92, for experienced neglect α_mother_ = .86 and α_father_ = .85, for perpetrated abuse α_child1_ = .82 and α_child2_ = .81, and for perpetrated neglect α_child1_ = .62 and α_child2_ = .61. Occurrence of maltreatment is reported in S1 and S3 Tables and in the Supplementary Material.

To create an *experienced* maltreatment score, for child and parent report, four scale scores (*Emotional Abuse*, *Physical Abuse*, *Emotional Neglect* and *Physical Neglect*) were calculated from participants’ self-reported experienced maltreatment and from mother and father self-reported perpetrated maltreatment towards that particular participant. For participants’ self-reported maltreatment (i.e., child report), scale scores were comprised of the highest score for father or mother (e.g., the highest score of *Emotional Abuse by father* or *Emotional Abuse by mother* was used for the score on the scale *Emotional Abuse*). If participants had more than one mother or father figure, they were instructed to report on the mother or father figure that they spent most time with growing up. Next, an overall *Abuse* score was comprised by averaging Emotional and Physical Abuse, and an overall *Neglect* score was comprised by averaging Emotional and Physical Neglect. The same scale scores were computed separately for mother and father self-reported perpetrated maltreatment towards that particular participant (i.e., parent report). This resulted in information from three informants to be combined for experienced maltreatment: father, mother and child score (highest score for father or mother).

To create a *perpetrated* maltreatment score for child and parent report, per scale and child, averages were computed. If multiple children reported on one parent or a parent reported on multiple children, the highest score per scale was included. We chose to combine the individual child reports, because a number of parents (*n* = 34) had one child. As a result, there were only two informants to be combined for perpetrated maltreatment.

Because the distribution of the CTS data was skewed to the right, scores were log-transformed and then multiplied by 10 to scale up the variance. There was one outlier *(n* = 1), which was winsorized, i.e., the difference between the two next highest values was added to the next highest value with standardized value < 3.29 [44] to fit the distribution.

### Preparatory analyses

#### Multiple imputation

Missing values were imputed by means of multiple imputation (MI) with the package ‘*mice*’ [45] in R [46]. In MI, missing values are estimated several times, resulting in several complete versions of the incomplete dataset. Each of these datasets are then analyzed using the statistical procedure of interest, and the results are combined using specific combination procedures that take into account the variation of the imputed values in the standard errors and *p*-values. MI has the advantage that no information is thrown away, and that at the same time uncertainty of the missing data is taken into account in the statistical analysis (e.g., [45,47]). The package ‘*mice*’ imputes incomplete multivariate data by chained equations (MICE [45]). The data were imputed 50 times incorporating both predictors and auxiliary variables, i.e. variables that are not part of the model, but that are correlated with the variables in the model. Auxiliary variables were household chaos (Household Chaos questionnaire [48]), number of siblings, non-verbal intelligence (Raven’s Standard Progressive Matrices [49]), attachment styles (Experiences in Close Relationships Questionnaire (ECR-RS: [50]), internalizing and externalizing problems (Child Behavioral Checklist (CBCL: [27]), Youth Self Report (YSR: [51]) & Adult Self Report (ASR: [52])), and eligibility (i.e., participated, declined or non-eligible). To improve our prediction model we included all participants from G2 and G3 to estimate experienced maltreatment, accumulating in a sample size of *n* = 335. Twenty percent of all values were missing (range: 0 to 54%). The majority of missing values were a result of one parent not participating. Self-report data on experienced maltreatment was complete. In 65% of the cases at least two reporters on maltreatment were available. In 40% of the cases three reporters on maltreatment were available, meaning that both parents participated. We used predictive mean matching (PMM: [53]) as multiple-imputation method. This method borrows an observed value from a donor with a similar predictive mean, so that imputed values never fall outside the range of the variable, or assume any other values that do not appear in the observed part of the variable. Autocorrelation function (ACF) plots revealed that all imputations converged (for a description of these plots see [54]). In addition, the correlations between variables were approximately the same in the imputed datasets (see Supplementary Material S3 Table) compared to the non-imputed dataset (see Supplementary Material S4 Table). Further analyses were conducted in SPSS version 23.

#### Informant agreement

We examined the absolute agreement between the different informants for experienced abuse and neglect separately by calculating the intraclass correlation coefficients (ICC (3,k), single measures, absolute agreement, see [55]). ICC (3,k) was employed with experienced abuse and neglect of each target (i.e., the child) being rated by three reporters (i.e., mother, father, child). Intraclass correlations were computed for father-child, mother-child, and father-mother pairs separately. ICC’s were averaged across imputed data sets. As shown in Table 1, agreement among all informants was modest (ICCs ≤ .35). The lowest level of agreement was found between MR and CR for neglect, whereas the highest level of agreement was found between father and mother report for neglect, implying parents reported relatively similar regarding neglectful behavior.

**Table 1:** Concordance between different informants of abuse and neglect.

	ICC (3,*k*)		
	Father-child	Mother-child	Father-mother
Abuse	0.32	0.28	0.31
Neglect	0.14	0.05	0.35

### Principal component analyses (PCA)

We then combined the different informants on experienced child abuse and neglect, by including father, mother and child scores in a principal component analysis (PCA; see [33] for a similar approach). Generalized procrustes analysis (GPA; [56]) was used as a method to combine the results of PCA in multiple imputation (see [57] for a description of this method in the context of multiple imputation). In line with previous studies using a factor-analytic approach to disaggregate variance from different informants [12,33] we set the number of factors to be extracted in the PCA equal to the number of informants (three in our case). The pooled component coefficients of each type of informant (mother/father/child) were then multiplied with the standardized original scores of each participant and summed up to obtain the component scores. Further, these component scores were correlated with perpetrated abuse and neglect. The results of the PCA with three higher-order factor scores for experienced abuse and neglect are presented in S5 Table of the Supplementary Material.

#### Abuse

For abuse, the first component–labeled *Reporter convergence–*showed high positive component loadings for all informants and indicates the convergent view of mother, father and child on the occurrence of abuse. This component explained 55% of the variance in the occurrence of abuse. The second component–*Mother report–*was defined by a high component loading for mother-reported abuse and negative component loadings for child- and father reported abuse. This component explained 24% of the variance in abuse. The third component was labeled *Father versus child report* because of a high component loading for father reported abuse, a negative component loading for child reported abuse and a component loading close to zero for mother reported abuse. This component explained 20% of the variance in the occurrence of abuse.

#### Neglect

For neglect, the first component–also labeled *Reporter convergence–*showed positive component loadings for all informants and indicates the convergent view of mother, father and child on the occurrence of neglect. This component explained 49% of the variance in the occurrence of neglect. The second component represented *Child report* since it was defined by a high positive component loading for child reported neglect and negative component loadings for mother reported and father reported neglect. This component explained 31% of the variance in the occurrence of neglect. The third component was interpreted as *Mother versus father report*, and showed a relatively high positive component loading for mother reported neglect, a negative loading for father report and a relatively low positive component loading for child reported neglect. This component explained 20% of the variance in neglect.

For perpetrated abuse and neglect there were only two informants (parents/children), which made PCA unnecessary. Component scores using a PCA on two items would give equivalent results as averaging the scores, since each item will get the same weight/loading in the PCA. Scores of parents and children were therefore averaged to create a perpetrated abuse and neglect score.

### Intergenerational transmission of child abuse and neglect

First, we tested intergenerational transmission with two common approaches using multiple hierarchical regression analyses: a) intergenerational transmission from the perspective of one reporter: regression analyses with self-reported perpetrated maltreatment as dependent variable and self-reported experienced maltreatment as continuous predictor (Design 1, Fig 1) and b) intergenerational transmission from the perspective of different reporters from each generation: regression analyses with self-reported experienced maltreatment of G3 (as indicator of G2 perpetrated maltreatment) as dependent variable and self-reported experienced maltreatment of their parents (G2) as a continuous predictor (Design 2, Fig 1). Analyses were performed separately for abuse and neglect. In a next step, two multiple hierarchical regression analyses were conducted for the multi-informant scores of perpetrated abuse and neglect (for details see *Measures* section), with the PCA component scores of abuse and neglect as continuous predictors to determine whether a component explained additional variance in perpetrated abuse and neglect beyond variance explained by the other components.

Gender, age and household socio-economic status (SES) were added as covariates in a first step in all regression analyses. In addition, the other type of maltreatment (i.e., abuse or neglect) was included in a last step to test whether the effects of abuse and neglect were unique. Pooled coefficients were provided by SPSS. We used the following formula to convert the unstandardized coefficients to standardized coefficients: Beta_j_ = B_j_ *(SD(X_j_)/SD(Y)). For pooling the point estimates of *R*^2^ the average across imputed data sets was calculated, and combination rules of [47] were used for testing the significance of *R*^2^ and Δ*R*^2^. See [58] for details on these procedures.

## Results

Descriptive statistics and correlations of study variables for the non-imputed study variables are presented in S3 Table in the Supplementary Material. Correlations for imputed study variables are presented in S4 Table in the Supplementary Material. There was significant reporter correlation within experienced and perpetrated abuse and perpetrated neglect (range: *r*(190) = .27 - .36, *p* < .05). For experienced neglect, FR correlated with both CR (*r*(190) = .24, *p* = .042) and MR (*r*(190) = .40, *p* = .001) but CR and MR were not significantly correlated (*r*(190) = .13, *p* = .171). According to CR (*r*(190) = -.17, *p* = .027) and parent report (PR; (*r*(190) = -.21, *p* = .004), fathers were more neglectful than mothers. Experienced abuse (*r*(190) = .20, *p* = .005) and neglect (*r*(190) = .34, *p* < .001) increased with age (CR). Higher household SES was associated with more PR perpetrated neglect (*r*(190) = .18, *p* = .013).

### Intergenerational transmission of abuse and neglect

Further, we tested whether participants from G2 (*n* = 191; including five G3 participants who also reported on perpetrated maltreatment) were more likely to perpetrate maltreatment if they had been maltreated during their childhood in hierarchical regression models for abuse and neglect separately.

#### Intergenerational transmission from the perspective of one reporter

Results of the hierarchical regressions for abuse and neglect from the perspective of one reporter are presented in Table 2. Abuse, age, gender and SES were included in the first step and explained 2% of the variance in self-reported perpetrated abuse. None of the covariates were significantly related to self-reported perpetrated abuse. In the next step, self-reported experienced abuse was included, which increased the explained variance of the model significantly with 23% (Δ*R*^2^ *=* 0.23, *F*(1,185) = 57.20, *p* < .001). Self-reported experienced abuse was significantly positively associated with self-reported perpetrated abuse (β = 0.47, *p* < .001), indicating intergenerational transmission of abuse when viewed from the perspective of one reporter. Results remained the same when including self-reported experienced neglect as a predictor at the last step (β of self-reported perpetrated abuse remained 0.47), meaning that self-reported experienced abuse was uniquely associated with self-reported perpetrated abuse.

**Table 2:** Hierarchical regression analyses for abuse and neglect testing intergenerational transmission from the perspective of one reporter.

		*B*	*SE(B)*	β	*t*	Sig. (*p*)	*F*	Sig. (*p*)	*R*^2^	Δ*R*^2^
Dependent variable: Perpetrated Abuse										
Step 1							01.38	< .25	02%	
	Gender	-0.02	0.12	-0.01	-0.19	< .85				
	Age	-0.00	0.01	-0.00	-0.10	< .92				
	SES	-0.10	0.08	-0.07	-1.19	< .23				
Step 2							15.61	< .001	25%	23%
	Experienced Abuse	-0.36	0.05	-0.47	-7.56	< .001				
Dependent variable: Perpetrated Neglect										
Step 1							08.16	< .001	12%	
	Gender	-0.40	0.14	-0.17	-2.94	< .001				
	Age	-0.01	0.01	-0.37	-1.52	< .13				
	SES	-0.27	0.10	-0.15	-2.81	< .01				
Step 2							10.59	< .001	19%	07%
	Experienced Neglect	-0.21	0.05	-0.28	-3.99	< .001				

*Note*. The displayed coefficients of the variables in Step 1 and 2 represent the values after inclusion of variables in Step 3. Persp. = perspective

For neglect the same steps were followed. Covariates included in the first step explained 12% of the variance in self-reported perpetrated neglect. Gender (β = -0.17, *p* = .014), age (β = 0.37, *p* = .002) and household SES (β = 0.15, *p* = .025) were significantly associated with self-reported perpetrated neglect, indicating that males, older participants and participants from households with a higher SES reported more perpetrated neglect towards their child(ren). Including self-reported experienced neglect in a second step increased the explained variance of the model with 7% (Δ*R*^2^ *=* 0.07, *F*(1,183) = 15.93, *p* < .001). Self-reported experienced neglect was significantly positively associated with self-reported perpetrated neglect (β = 0.28, *p* < .001), indicating intergenerational transmission of neglect from the perspective of one reporter. Self-reported experienced neglect remained significantly associated with self-reported perpetrated neglect (β = 0.31, *p* < .001) after including self-reported abuse in a third step, meaning that self-reported experienced neglect was uniquely associated with self-reported perpetrated neglect

#### Intergenerational transmission from the perspective of different reporters from each generation

Results of the hierarchical regressions for abuse and neglect from the perspective of different reporters from each generation are presented in Table 3. For abuse, age, gender and SES included in a first step explained 2% of the variance in G2 perpetrated abuse *reported by* G3. None of the covariates were significantly associated with G2 perpetrated abuse. In a next step, experienced abuse reported by G2 was included, which increased the explained variance with 6% (Δ*R*^2^ *=* 0.06, *F*(1,173) = 11.13, *p* = .001). Experienced abuse reported by G2 was significantly positively associated with G2 perpetrated abuse (β = 0.27, *p* = .001), indicating that there was intergenerational transmission of abuse when viewed from the perspective of different reporters from each generation. The association between experienced abuse reported by G2 and G2 perpetrated abuse remained significant (β = 0.25 *p* = .006) after controlling for experienced neglect reported by G2, indicating that experienced abuse reported by G2 was uniquely associated with G2 perpetrated abuse.

**Table 3:** Hierarchical regression analyses for abuse and neglect testing intergenerational transmission using different reporters of experienced maltreatment for the perspective of each generation.

		*B*	*SE(B)*	β	*t*	Sig. (*p*)	*F*	Sig. (*p*)	*R*^2^	Δ*R*^2^
Dependent variable: Perpetrated Abuse										
Step 1							.82	.49	2%	
	Gender	-0.05	0.15	-0.03	-0.35	.73				
	Age	-0.01	0.01	-0.13	-0.91	.37				
	SES	-0.00	0.11	-0.02	-0.02	.98				
Step 2								.02	8%	6%
	Experienced Abuse	-0.20	0.06	-0.27	-3.34	.00				
Dependent variable: Perpetrated Neglect										
Step 1							2.93	.04	6%	
	Gender	-0.41	0.17	-0.20	-2.44	.02				
	Age	-0.01	0.01	-0.13	-0.75	.45				
	SES	-0.22	0.12	-0.15	-1.78	.08				
Step 2								.07	6%	0%
	Experienced Neglect	-0.01	0.06	-0.01	-0.07	.94				

*Note*. The displayed coefficients of the variables in Step 1 and 2 represent the values after inclusion of variables in Step 3. Persp. = perspective

For neglect, covariates included in a first step explained 6% of the variance in G2 perpetrated neglect *reported by* G3. Of the covariates, only gender of G2 was significantly associated with experienced neglect reported by G3 (β = -0.20, *p* = .015), indicating that children reported to be neglected by fathers more often. Experienced neglect reported by G2 was included in a next step, which did not significantly increase the explained variance (Δ*R*^2^ *=* 0.00, *F*(1,156) = 0.01, *p* = .941). In addition, experienced neglect reported by G2 was not significantly associated with experienced neglect reported by G3 (β = 0.01, *p* = .941). The association remained non-significant (β = -0.02, *p* = .873) when controlled for experienced abuse reported by G2, indicating no unique effects of experienced neglect reported by G2 on G2 perpetrated neglect.

#### Intergenerational transmission using a multi-informant approach

Results of the hierarchical regressions for abuse and neglect using a multi-informant approach are presented in Table 4. For abuse, age, gender and household SES were included in the first step. This model explained 3% of the variance in the multi-informant scores of perpetrated abuse by G2 (parent and child report averaged). None of the covariates were significantly related to perpetrated abuse. In the next step, the component *Reporter convergence* was included. The explained variance in the multi-informant scores of perpetrated abuse increased significantly with 9% (Δ*R*^2^ = 0.09, *F*(1,103) = 12.51, *p* < .001). *Reporter convergence* was positively and significantly associated with perpetrated abuse (β = 0.30, *p* < .001), supporting intergenerational transmission based on agreement between all reporters. In the third step the components *Mother report* and *Father versus child report* were added, which increased the explained variance of the model with 12% (Δ*R*^2^ *=* 0.12, *F*(2,103) = 4.99, *p* = .009). *Father versus child report* was significantly and negatively associated with perpetrated abuse (β = -0.34, *p* = .001) indicating that the discrepancy in father and child reports on experienced abuse improved the prediction of perpetrated abuse beyond the component *Reporter convergence*. *Mother report* was not significantly associated with perpetrated abuse (β = -0.05, *p* = .640). Associations of the components *Convergence* and *Father versus child report* of abuse with perpetrated abuse remained significant (β = 0.30, *p* < .001 and β = -0.34, *p* = .003, respectively) when including the component *Reporter convergence* of Neglect in a fourth step. This indicates that the components *Convergence* and *Father versus child report* of experienced abuse were uniquely associated with perpetrated abuse, when controlling for neglect.

**Table 4:** Hierarchical regression analyses for abuse and neglect using a multi-informant approach.

		*B*	*SE(B)*	β	*t*	Sig. (*p*)	*F*	Sig. (*p*)	*R*^2^	Δ*R*^2^
Dependent variable: Perpetrated Abuse										
Step 1							1.40	< .24	03%	
	Gender	-0.01	0.11	-0.00	-0.042	= .97				
	Age	-0.01	0.01	-0.11	-0.78	= .44				
	SES	-0.06	0.08	-0.05	-0.693	= .49				
Step 2							4.78	< .001	12%	09%
	Reporter convergence	-0.14	0.04	-0.30	-3.68	< .001				
Step 3							5.13	< .001	24%	12%
	Mother report	-0.06	0.13	-0.05	-0.47	= .64				
	Father vs. child report	-0.42	0.13	-0.34	-3.27	= .001				
Dependent variable: Perpetrated Neglect										
Step 1							3.54	< .02	05%	
	Gender	-0.40	0.13	0.23	-3.13	0.002				
	Age	-0.00	0.01	-0.14	-0.04	= .97				
	SES	-0.02	0.09	-0.02	-0.23	= .82				
Step 2							2.31	< .06	06%	01%
	Reporter convergence	-0.04	0.06	-0.06	-0.64	0.53				
Step 3							1.41	< .21	10%	04%.
	Child report	-0.14	0.09	-0.15	-1.56	= .12				
	Mother vs. father report	-0.12	0.20	-0.08	-0.58	= .56				

*Note*. The displayed coefficients of the variables in Step 1 and 2 represent the values after inclusion of variables in Step 3.

For neglect the same steps were followed. Covariates were included in the first step and explained 5% of the variance in the multi-informant scores of perpetrated neglect (parent and child report combined). Of the covariates, only gender was significantly related to perpetrated neglect (β = -0.23, *p* = .002), indicating that on average men perpetrated more neglect than women. The component *Reporter convergence* was included in the next step. There was no significant increase in explained variance (Δ*R*^2^ = 0.01, *F*(1,81) = 0.01, *p* = .970) and *Reporter convergence* was not significantly associated with perpetrated neglect (β = 0.06, *p* = .526), suggesting that intergenerational transmission of neglect as observed by all informants was not supported. The components *Child report* and *Mother versus father report* were added in the third step. There was no significant increase in explained variance of this model (Δ*R*^2^ = 0.04, *F*(2,90) = 0.66, *p* = .52). Both *Child report* (β = 0.15, *p* = .119) and *Mother versus father report* (β = 0.08, *p* = .560) were not significantly associated with perpetrated neglect, indicating that the divergent reports on experienced neglect did not contribute to the prediction of perpetrated neglect beyond the component *Reporter convergence*. When including the component *Convergence* of abuse in a fourth step, all associations of the components of neglect with perpetrated neglect remained non-significant (*Reporter convergenc*e: β = 0.08, *p* = .434, *Child report*: β = 0.15, *p* = .105, *Mother versus father report*: (β = 0.09, *p* = .520). This indicates that convergence of informants and unique perspectives of informants on experienced neglect were not uniquely associated with perpetrated neglect. To correct for the nested family structure, we replicated these results using a multi-level analysis (S6–S8 Tables in the Supplementary Material).

## Discussion

The three-generational multi-informant design of the 3G Parenting Study enabled us to investigate intergenerational transmission of child maltreatment (ITCM) using multiple sources of information on abuse and neglect, i.e., mothers, fathers and children. Our study offers new insight into reporter effects on ITCM: a) intergenerational transmission of abuse was consistently found across approaches–from the perspective of one reporter, from the perspective of different reporters from each generation and using the multisource approach, b) father versus child report contributed significantly to the prediction of perpetrated abuse, and c) intergenerational transmission of neglect was only found using the perspective and data of one single reporter.

### Agreement and integration of different reports

In line with previous results on part of the sample [59] agreement between mothers, fathers and children on abuse and neglect was modest. The lowest agreement was found between children and parents on neglect, whereas the highest agreement was between fathers and mothers on neglect. In the study of [22] it was suggested that there might be a gap between what parents feel (i.e., love) and what they convey (i.e., tell your children you love them), explaining the low agreement between parents and children. In addition, discrepancies between parents and children on neglect may occur due to changing beliefs across generations about appropriate parenting practices. Finally, abuse describes the presence of behavior whereas neglect describes the absence of behavior. It is possible to estimate the presence of behaviors without judging whether that behavior was adequate (e.g., My mother/ father shouted, yelled or screamed at me), whereas estimating the absence of behavior usually requires a judgment whether the behavior should have been present (e.g., My father/mother was not able to make sure that I got to the doctor or hospital when needed). The measurement of neglect might therefore be more subjective than the measurement of abuse. Considering the retrospective nature of the measurement, the absence of behavior may more difficult to recall than presence of behavior [60]. Parent couples, however, reported fairly similar neglectful behavior. This might be explained by the fact that many parents share attitudes and beliefs about appropriate parenting practices that guide their behavior [61].

Results of the PCA were in line with Kraemer’s prediction that all informants should contribute to the first component in the same direction (i.e., positive component loadings) if they are well selected. A useful component structure with three components was established in which the first component reflected the convergent reports of informants and the other components reflected unique perspectives on the occurrence of maltreatment. Since parents reported only on their own behavior, it is difficult to determine whether convergence reflects similarity in behavior or similarity in perception of behavior. In line with a previous study that applied this approach to maltreatment [33], convergent reports explained most of the variance (around 50%) in abuse and neglect, despite challenges in querying children and caregivers on this subject, distorted memories [18] or reluctance to report on maltreatment [15,16]. More importantly, including multiple perspectives may increase validity, since random error and systematic bias is reduced. It should be noted that child report contributed less to convergence for neglect than for abuse, which confirms the results of the intra-class correlations that revealed low agreement between children and parents on the occurrence of neglect.

For convergence on abuse, we found very similar weights for child, father, and mother report (i.e., 0.76, 0.75, and 0.72 respectively). This means that the convergence score calculated in the current study is virtually equivalent to a mean score of the three reporters. Thus, for researchers primarily interested in a combined multi-informant score of abuse a mean score may suffice. However, replication of this finding is warranted. Combining neglect scores from parents and children may be more complex as there might be more disagreement between them. Ultimately, the specific research question should guide decisions on the method of combining maltreatment reports. In the current study we chose a data-driven approach of combining reports (i.e., PCA) but for research questions that underlie a hypothetical process or construct, a theory-driven approach might be better suited.

### Intergenerational transmission of child maltreatment

With regard to the predictive strength of the components in ITCM, the convergent perspective of experienced abuse predicted perpetrated abuse, indicating intergenerational transmission of abuse when multiple perspectives are combined. Intergenerational transmission of abuse was also found using more conventional approaches: i.e., reports of only one informant or reports of different reporters from each generation (see Fig 1 for an illustration). This suggests that in the present study evidence of intergenerational transmission of abuse was found independent of the source of information. Nevertheless, the approach of testing ITCM can affect the magnitude of the transmission. If only one perspective was included the explained variance was the highest (23%) compared to including multiple informants (< 10%). Thus, reporter effects, such as distorted memories or reluctance to report on incidences of maltreatment, may play a role but cannot fully explain the intergenerational transmission of abuse.

The component father versus child report on experienced abuse explained additional variance in the perpetration of abuse above and beyond the convergent reports of all informants. This indicates that differences in reports of G1 fathers (i.e., grandfathers) and G2 children on abuse experienced by G2 were predictive of G2 perpetrated abuse towards G3. The transmission was strongest when children reported higher levels of abuse than fathers. Possibly, this indicates that sharing a similar perspective might buffer the negative effects of maltreatment to some extent. Our findings thus provide support for the relevance of including fathers in research on ITCM, despite the fact that most studies on ITCM focused only on mothers [6,13,62]. Considering child maltreatment incidence, there seems little reason to exclude fathers in ITCM research. Even though sex differences exist in child maltreatment prevalence rates, research clearly indicates that both boys and girls may be victims of child maltreatment and that fathers just as mothers may be perpetrators [63,64]. Results of the current study showed that fathers compared to mother were more likely to neglect their children across approaches that were used to estimate ITCM, emphasizing the relevance of studying predictors of neglect perpetrated by fathers. Finally, including fathers in research on maltreatment may be especially important since fathers’ involvement in child care has continuously increased in many Western countries the past few decades [65].

Regarding neglect, we found evidence for intergenerational transmission of neglect when using the perspective of one reporter, i.e., self-reported experienced neglect predicted self-reported perpetrated neglect. This confirms our first hypothesis: ITCM was found for both abuse and neglect when one informant reports about experienced and perpetrated maltreatment. Yet, transmission of neglect was not confirmed with our component-analytical approach or when reports of different informants from each generation were used, i.e., experienced neglect reported by G2 was not significantly associated with G2 perpetrated neglect reported by G3. Intergenerational transmission of neglect thus seemed to disappear when reports of multiple informants are used. It has repeatedly been discussed that bias is likely in studies on ITCM when a single reporter for the independent and dependent variables is used [8,13]. For example, parents may overreport victimization as a means to defend their own perpetrating behavior [32]. Conversely they may underreport their victimization to protect those who maltreated them or a desire to forget the victimization [16]. Hence, it can be called into question whether neglectful behavior is truly transmitted from one generation to the next, or whether evidence of transmission is driven by dependency of the perceptions of one person. This may be particularly relevant considering the modest agreement between parents and children discussed earlier.

### Dealing with missing data

Our three-generational design enabled us to include reports from parents and children of both experienced and perpetrated maltreatment in estimating ITCM, thereby reducing reporter bias. However, recruiting families is challenging and missingness due to family members who do not participate is quite high. Yet, we would argue that the risk of missingness should not discourage researchers from using multi-informant methods to estimate and investigate ITCM, since modern techniques for handling missing data such as Multiple Imputation (MI) offer compelling solutions. Many statisticians consider MI the “gold standard” for handling missing data, because it produces less bias than other typical methods [66,67]. MI has been shown to be statistically sound especially for large percentages of missing values as wider confidence intervals will be generated for variables with more missing data, avoiding the risk of false positives [67].

While the use of MI appears to be on the rise [68] it is far from being standard practice. Several reviews of the handling of missing data in various fields showed that only very few studies used MI [69,70]. This might be problematic in the context of child maltreatment as our results show that parents who did not participate may differ systematically from those who participated in terms of abuse and neglect. Specifically–according to their children–parents who did not participate were more likely to neglect but less likely to abuse their children. It is not difficult to imagine that neglectful parents are also less likely to participate with their children in research. The finding that parents with higher scores on abuse did not refrain from participation in the study is promising and adds to the reliability of our findings. We were able to use this and other information in our prediction model for MI. As such, MI offers a useful solution for systematic missingness. Imputations converged across datasets and correlations between variables were approximately similar for non-imputed and multiply imputed data. Findings of the present study thus suggest that having complete families is not required when estimating ITCM based on integrated data from multiple informants.

### Limitations

The current study is not without limitations. First, the majority of the participants were adults and reported about childhood events retrospectively. Findings of a recent meta-analysis suggests limited overlap between retrospective and prospective report [71]. The retrospective nature in reporting may increase measurement error and false negatives due to denial [60] or memory loss [72]- especially when the experience happened a long time ago (as was the case for some adults).Studies with larger samples size to estimate age differences reliably or using a prospective design may provide insight into the effects of the timing of the reports on estimates of ITCM. Currently, most ITCM studies with prospective designs have a short follow-up period [13]. As a result they generally only cover the first period of childhood–potentially missing maltreatment with a later onset. A prospective study following three generations may not be feasible and a retrospective design with multiple reporters might be the second best option. The design of the current study allowed us to cover parenting experiences across the entire span of growing up for most participants. Lastly, bias due to retrospective reporting should not be given too much weight as research has shown that false positives in maltreatment research are rare and associations with psychopathology are comparable for retrospective and prospective reports [72,73]. Some evidence suggests that associations between retrospective reports of maltreatment and psychopathology might be stronger [74]. Another limitation of the current study was that the sample is not fully representative of the general population, because the majority of the participants in our sample had a Caucasian background. In addition, it is important to recognize that parents only report on their own behavior allowing for different interpretations of convergence of mother and father report. While combining multiple reports on child maltreatment adds valuable information, an even more comprehensive picture could be gained by asking parents also about their partners’ behavior. This would also support disentangling perceptions from behavior.

### Implications

With regard to policy and clinical practice, our data suggest that including multiple informants in research on ITCM may be valuable for obtaining a comprehensive picture of maltreatment incidences and their consequences for parenting behavior in the next generation. Moreover, combining multiple perspectives in prevalence studies may reduce random error and systematic bias in prevalence estimates. Conversely, whenever informants differ, professionals should carefully attend to the exact pattern of reports. Father reports may turn out to be especially informative regarding risk for ITCM, but the precise implications should be explored further. Including both mothers and fathers should become standard practice in child maltreatment research.

## Conclusion

In conclusion, this is one of very few ITCM studies that uses multi-informant reports and includes fathers. We found evidence of intergenerational transmission of abuse using three different methods (see Fig 1). In addition to convergent reports of mother, father and child on experienced abuse, father vs. child report on experienced abuse uniquely contributed to the prediction of perpetrated abuse, highlighting the importance of including fathers in research on ITCM. Overall, despite the significant association between experienced and perpetrated abuse, it is important to keep in mind that most abused parents do not go on to abuse their own children. For neglect, intergenerational transmission was only found when the same individual reported on experienced and perpetrated neglect calling into question whether there is “actual” transmission of neglect. Neglect represents the absence of behavior and is a more abstract construct which might be difficult to assess. Although research has shifted more towards studying neglect, continued efforts are needed to improve our understanding of the assessment, precursors and sequelae of neglect.

## Supporting information

S1 Text The 3G Parenting Study. (DOCX)

S2 Text Informant agreement. (DOCX)

S1 Table Occurrence of self-reported experienced emotional and physical abuse and neglect. (DOCX)

S2 Table Occurrence of self-reported perpetrated emotional and physical abuse and neglect. (DOCX)

S3 Table Component loadings of the Principal Component Analysis (PCA) for maltreatment by multiple informants. (DOCX)

S4 Table Correlations of the pooled observed variables (*n* = 192). (DOCX)

S5 Table Summary of Correlations, Means, and Standard Deviations of non-imputed observed variables (N = 28–335). (DOCX)

S6 Table Stepwise multilevel model for abuse and neglect testing intergenerational transmission from the perspective of one reporter. (DOCX)

S7 Table Stepwise multilevel model for abuse and neglect testing intergenerational transmission using different reporters of experienced maltreatment for the perspective of each generation. (DOCX)

S8 Table Stepwise multilevel model for abuse and neglect using a multi-informant approach. (DOCX)
