# Correction: Intergenerational transmission of child maltreatment using a multi-informant multi-generation family design

**DOI:** 10.1371/journal.pone.0232792

**Published:** 2020-04-30

**Authors:** Renate S. M. Buisman, Katharina Pittner, Marieke S. Tollenaar, Jolanda Lindenberg, Lisa J. M. van den Berg, Laura H. C. G. Compier-de Block, Joost R. van Ginkel, Lenneke R. A. Alink, Marian J. Bakermans-Kranenburg, Bernet M. Elzinga, Marinus H. van IJzendoorn

There are errors in the values presented in Tables 2, 3 and 4. A number of symbols are present that are incorrect. Please see the correct Tables 2, 3 and 4 here.

**Table 2:** Hierarchical regression analyses for abuse and neglect testing intergenerational transmission from the perspective of one reporter.

	*B*	*SE(B)*	β	*t*	Sig. (*p*)	*F*	Sig. (*p*)	*R*^2^	Δ*R*^2^
Dependent variable: Perpetrated Abuse									
Step 1						1.38	.25	2%	
Gender	-0.02	0.12	-0.01	-0.19	.85				
Age	0.00	0.01	0.00	0.10	.92				
SES	-0.10	0.08	-0.07	-1.19	.23				
Step 2						15.61	< .001	25%	23%
Experienced Abuse	0.36	0.05	0.47	7.56	< .001				
Dependent variable: Perpetrated Neglect									
Step 1						8.16	< .001	12%	
Gender	-0.40	0.14	-0.17	-2.94	< .001				
Age	0.01	0.01	0.37	1.52	.13				
SES	0.27	0.10	0.15	2.81	.01				
Step 2						10.59	< .001	19%	7%
Experienced Neglect	0.21	0.05	0.28	3.99	< .001				

*Note*. The displayed coefficients of the variables in Step 1 and 2 represent the values after inclusion of variables in Step 3. Persp. = perspective

**Table 3:** Hierarchical regression analyses for abuse and neglect testing intergenerational transmission using different reporters of experienced maltreatment for the perspective of each generation.

	*B*	*SE(B)*	β	*t*	Sig. (*p*)	*F*	Sig. (*p*)	*R*^2^	Δ*R*^2^
Dependent variable: Perpetrated Abuse									
Step 1						0.82	.49	2%	
Gender	0.05	0.15	0.03	0.35	.73				
Age	0.01	0.01	0.13	0.91	.37				
SES	0.00	0.11	-0.02	-0.02	.98				
Step 2							.02	8%	6%
Experienced Abuse	0.20	0.06	0.27	3.34	< .001				
Dependent variable: Perpetrated Neglect									
Step 1						2.93	.04	6%	
Gender	-0.41	0.17	-0.20	-2.44	.02				
Age	-0.01	0.01	-0.13	-0.75	.45				
SES	-0.22	0.12	-0.15	-1.78	.08				
Step 2							.07	6%	0%
Experienced Neglect	0.01	0.06	0.01	0.07	.94				

*Note*. The displayed coefficients of the variables in Step 1 and 2 represent the values after inclusion of variables in Step 3. Persp. = perspective

**Table 4:** Hierarchical regression analyses for abuse and neglect using a multi-informant approach.

	*B*	*SE(B)*	β	*t*	Sig. (*p*)	*F*	Sig. (*p*)	*R*^2^	Δ*R*^2^
Dependent variable: Perpetrated Abuse									
Step 1						1.40	.24	3%	
Gender	0.01	0.11	0.00	0.04	.97				
Age	0.01	0.01	0.11	0.78	.44				
SES	-0.06	0.08	-0.05	-0.69	.49				
Step 2						4.78	.001	12%	9%
Reporter convergence	0.14	0.04	0.30	3.68	< .001				
Step 3						5.13	< .001	24%	12%
Mother report	-0.06	0.13	-0.05	-0.47	.64				
Father vs. child report	-0.42	0.13	-0.34	-3.27	.001				
Dependent variable: Perpetrated Neglect									
Step 1						3.54	.02	5%	
Gender	-0.40	0.13	0.23	-3.13	.002				
Age	0.00	0.01	0.14	0.04	.97				
SES	0.02	0.09	0.02	0.23	.82				
Step 2						2.31	.06	6%	1%
Reporter convergence	0.04	0.06	0.06	0.64	.53				
Step 3						1.41	.21	10%	4%.
Child report	0.14	0.09	0.15	1.56	.12				
Mother vs. father report	0.12	0.20	0.08	0.58	.56				

*Note*. The displayed coefficients of the variables in Step 1 and 2 represent the values after inclusion of variables in Step 3.
