# Supplementary material for: Intergenerational transmission of child maltreatment using a multi-informant multi-generation family design
Source: PLoS One. 2020 Mar 12;15(3):e0225839. doi: 10.1371/journal.pone.0225839 (PMC7067458; doi:10.1371/journal.pone.0225839)
Supplement: S1 Text — (DOCX) [file pone.0225839.s001.docx]

**S1 Text.** The 3G Parenting Study

The 3G Parenting Study is a cross-sectional three-generational extended family study on the interplay of methodological (e.g., reporter effects), genetic, and environmental factors involved in the intergenerational transmission of parenting styles, stress and emotion regulation. We operationalized mechanisms of (intergenerational transmission of) child maltreatment at multiple levels: behavioral, physiological (autonomic and hormonal), neurological (brain morphology and activity), and with quantitative and molecular genetics.
