# Supplementary material for: Intergenerational transmission of child maltreatment using a multi-informant multi-generation family design
Source: PLoS One. 2020 Mar 12;15(3):e0225839. doi: 10.1371/journal.pone.0225839 (PMC7067458; doi:10.1371/journal.pone.0225839)
Supplement: S2 Text — (DOCX) [file pone.0225839.s002.docx]

**S2 Text. Informant agreement**

We examined the absolute agreement between the different informants for experienced abuse and neglect separately by calculating the intraclass correlation coefficients (ICC (3,k), single measures, absolute agreement, see Shrout & Fleiss, 1979). ICC (3,k) was employed with experienced abuse and neglect of each target (i.e., the child) being rated by three reporters (i.e., mother, father, child). Intraclass correlations were computed for father-child, mother-child, and father-mother pairs separately. ICC’s were averaged across imputed data sets. As shown in S4 Table, agreement among all informants was modest (ICCs ≤ .35). The lowest level of agreement was found between MR and CR for neglect, whereas the highest level of agreement was found between father and mother report for neglect, implying parents reported relatively similar regarding neglectful behavior.
