# Supplementary material for: Intergenerational transmission of child maltreatment using a multi-informant multi-generation family design
Source: PLoS One. 2020 Mar 12;15(3):e0225839. doi: 10.1371/journal.pone.0225839 (PMC7067458; doi:10.1371/journal.pone.0225839)
Supplement: S1 Table — (DOCX) [file pone.0225839.s003.docx]

**S1 Table.** **Occurrence of self-reported experienced emotional and physical abuse and neglect.^a, b^**

|  | Never | Once | More than once |
| --- | --- | --- | --- |
| Abuse | 14 (4%) | 11 (3%) | 310 (93%) |
| Physical Abuse | 72 (21%) | 41 (12%) | 222 (66%) |
| Emotional Abuse | 26 (8%) | 17 (5%) | 292 (87%) |
| Neglect | 12 (4%) | 3 (1%) | 320 (96%) |
| Physical Neglect | 215 (64%) | 24 (7%) | 96 (29%) |
| Emotional Neglect^c^ | 12 (4%) | 3 (1%) | 320 (96%) |

^a^Children reported about mother and father.
^b^Occurrences are based on items describing concrete parenting behaviors rather than the overall scales.
^c^ Note that four of the emotional neglect items were recoded. This means that participants who ‘never’ experienced emotional neglect, reported that they ‘(almost) always’ felt emotionally supported.
