# Supplementary material for: Intergenerational transmission of child maltreatment using a multi-informant multi-generation family design
Source: PLoS One. 2020 Mar 12;15(3):e0225839. doi: 10.1371/journal.pone.0225839 (PMC7067458; doi:10.1371/journal.pone.0225839)
Supplement: S2 Table — (DOCX) [file pone.0225839.s004.docx]

**S2 Table.** **Occurrence of self-reported perpetrated emotional and physical abuse and neglect^a^.**

|  | Never | Once | More than once |
| --- | --- | --- | --- |
| Abuse | 12 (5%) | 19 (8%) | 219 (88%) |
| Physical Abuse | 19 (8%) | 34 (14%) | 197 (79%) |
| Emotional Abuse | 61 (25%) | 43 (17%) | 146 (58%) |
| Neglect | 27 (11%) | 31 (12%) | 192 (77%) |
| Physical Neglect | 172 (69%) | 57 (23) | 21 (8%) |
| Emotional Neglect^b^ | 31 (12%) | 34 (14%) | 185 (74%) |

^a^ Parents reported about up to three children. As the number of children varied across parents, occurrence was based on the highest child score.

^b^ Note that four of the emotional neglect items were recoded. This means that participants who ‘never’ experienced emotional neglect, reported that they ‘(almost) always’ felt emotionally supported.
