# Supplementary material for: Intergenerational transmission of child maltreatment using a multi-informant multi-generation family design
Source: PLoS One. 2020 Mar 12;15(3):e0225839. doi: 10.1371/journal.pone.0225839 (PMC7067458; doi:10.1371/journal.pone.0225839)
Supplement: S3 Table — (DOCX) [file pone.0225839.s005.docx]

**S3 Table. Component loadings of the Principal Component Analysis (PCA) for maltreatment by multiple informants**

| Maltreatment | Component | | | | |
| --- | --- | --- | --- | --- | --- |
|  | Rep. convergence |  | Mother report |  | Father vs child report |
| Abuse |  |  |  |  |  |
| Child report | 0.76 |  | -0.29 |  | -0.58 |
| Father report | 0.75 |  | -0.36 |  | 0.55 |
| Mother report | 0.72 |  | 0.69 |  | 0.04 |
| Explained variance | 55% |  | 24% |  | 20% |
|  | Rep. convergence |  | Child report |  | Mother vs father report |
| Neglect |  |  |  |  |  |
| Child report | 0.51 |  | 0.84 |  | 0.21 |
| Father report | 0.81 |  | -0.10 |  | -0.57 |
| Mother report | 0.74 |  | -0.47 |  | 0.49 |
| Explained variance | 49% |  | 31% |  | 20% |
