# Supplementary material for: Intergenerational transmission of child maltreatment using a multi-informant multi-generation family design
Source: PLoS One. 2020 Mar 12;15(3):e0225839. doi: 10.1371/journal.pone.0225839 (PMC7067458; doi:10.1371/journal.pone.0225839)
Supplement: S4 Table — (DOCX) [file pone.0225839.s006.docx]

**S4 Table.** **Correlations of the pooled observed variables (*n* = 192)**

|  | Exp. Abuse CR | Exp. Abuse FR | Exp. Abuse MR | Perp. Abuse PR | Perp. Abuse CR | Exp. Neglect CR | Exp. Neglect FR | Exp. Neglect MR | Perp. Neglect PR | Perp. Neglect CR | Gender G2 | Age G2 |
| --- | --- | --- | --- | --- | --- | --- | --- | --- | --- | --- | --- | --- |
| Exp. Abuse FR | .39** |  |  |  |  |  |  |  |  |  |  |  |
| Exp. Abuse MR | .27** | .30* |  |  |  |  |  |  |  |  |  |  |
| Perp. Abuse PR | .50** | .21* | .29** |  |  |  |  |  |  |  |  |  |
| Perp. Abuse CR | .26** | -.05 | -.03 | .34** |  |  |  |  |  |  |  |  |
| Exp. Neglect CR | .48** | .22 | .09 | .24** | .17* |  |  |  |  |  |  |  |
| Exp. Neglect FR | .05 | .10 | .17 | -.04 | -.15 | .24* |  |  |  |  |  |  |
| Exp. Neglect MR | .03 | .17 | .34** | -.11 | -.19 | .13 | .40** |  |  |  |  |  |
| Perp. Neglect PR | .12 | .00 | .04 | .32** | .10 | .30** | .17 | .05 |  |  |  |  |
| Perp. Neglect CR | .04 | -.09 | -.03 | .18* | .29** | -.01 | -.17 | .02 | .36** |  |  |  |
| Gender G2 | -.05 | -.05 | .07 | -.03 | .00 | .05 | .01 | -.05 | -.21** | -.17* |  |  |
| Age G2 | .20** | .10 | .01 | .10 | .12 | .34** | -.14 | .00 | .25** | -.05 | -.14* |  |
| Household SES | -.04 | -.06 | -.14 | -.10 | .00 | -.09 | .01 | .11 | .18* | -.14 | -.04 | .08 |
| *Note*. CR = Child report; FR = Father report; MR = mother report; PR = Parent report; Exp. = maltreatment experienced by G2; Perp. = maltreatment perpetrated by G2; Gender: male coded as 1 and female coded as 2. Exp. Abuse FR indicate the abuse that was experienced by the child, but was perpetrated and reported by the father. The same applies to Exp. Abuse MR.  **p* < .05; ***p* < .01 | | | | | | | | | | | | |
