# Supplementary material for: Intergenerational transmission of child maltreatment using a multi-informant multi-generation family design
Source: PLoS One. 2020 Mar 12;15(3):e0225839. doi: 10.1371/journal.pone.0225839 (PMC7067458; doi:10.1371/journal.pone.0225839)
Supplement: S6 Table — (DOCX) [file pone.0225839.s008.docx]

**S6 Table. Stepwise multilevel model for abuse and neglect testing intergenerational transmission from the perspective of one reporter**

|  | Model 1 | Model 2 | Model 3 |
| --- | --- | --- | --- |
| Dependent variable: Perpetrated abuse | | | |
| Fixed effects | Coef (se) | Coef (se) | Coef (se) |
| Intercept | 1.57 (0.07)*** | 1.19 (0.41)** | 0.84 (0.36)** |
| Gender |  | -0.04 (0.13) | -0.02 (0.12) |
| Age |  | 0.01 (0.01) | 0.01 (0.01) |
| SES |  | -0.13 (0.09) | -0.10 (0.08) |
| Experienced Abuse |  |  | 0.36 (0.05)*** |
| Variance components |  |  |  |
| Individual level | 0.79 | 0.78 | 0.62 |
| Family level | 0.05 | 0.04 | 0.01 |
| Dependent variable: Perpetrated neglect | | | |
| Fixed effects | Coef (se) | Coef (se) | Coef (se) |
| Intercept | 1.61 (0.07)** | 1.13 (0.42)** | 1.10 (0.40)** |
| Gender |  | -0.35(0.14)* | -0.40 (0.01)** |
| Age |  | 0.02 (0.01)** | 0.01 (0.01) |
| SES |  | 0.22 (0.10)* | 0.27 (0.10)** |
| Experienced Neglect |  |  | 0.21 (0.05)*** |
| Variance components |  |  |  |
| Individual level | 1.00 | 0.89 | 0.82 |
| Family level | 0.00 | 0.00 | 0.00 |

*Note*. The unstandardized coefficients are represented. * *p* < .05, ** *p* < .01, ****p* < .001
