# Supplementary material for: Intergenerational transmission of child maltreatment using a multi-informant multi-generation family design
Source: PLoS One. 2020 Mar 12;15(3):e0225839. doi: 10.1371/journal.pone.0225839 (PMC7067458; doi:10.1371/journal.pone.0225839)
Supplement: S7 Table — (DOCX) [file pone.0225839.s009.docx]

**S7 Table**. **Stepwise multilevel model for abuse and neglect testing intergenerational transmission using different reporters of experienced maltreatment for the perspective of each generation**

|  | Model 1 | Model 2 | Model 3 |
| --- | --- | --- | --- |
| Dependent variable: Perpetrated abuse | | | |
| Fixed effects | Coef (se) | Coef (se) | Coef (se) |
| Intercept | 1.44 (0.10)*** | 0.64 (0.55) | 0.47 (0.55) |
| Gender |  | 0.09 (0.14) | 0.10 (0.14) |
| Age |  | 0.01 (0.01) | 0.01 (0.01) |
| SES |  | -0.01 (0.12) | 0.01 (0.12) |
| Experienced Abuse |  |  | 0.16 (0.06)** |
| Variance components |  |  |  |
| Individual level | 0.74 | 0.71 | 0.71 |
| Family level | 0.31 | 0.33 | 0.24 |
| Dependent variable: Perpetrated neglect | | | |
| Fixed effects | Coef (se) | Coef (se) | Coef (se) |
| Intercept | 2.18 (0.11)*** | 3.23 (0.57)*** | 3.23 (0.57)*** |
| Gender |  | -0.40 (0.15)** | -0.40 (0.15)** |
| Age |  | -0.01 (0.01) | -0.01 (0.01) |
| SES |  | -0.13 (0.13) | -0.13 (0.13) |
| Experienced Neglect |  |  | -0.01 (0.06) |
| Variance components |  |  |  |
| Individual level | 0.83 | 0.78 | 0.78 |
| Family level | 0.38 | 0.36 | 0.36 |

*Note*. The unstandardized coefficients are represented. * *p* < .05, ** *p* < .01, ****p* < .001
