# Supplementary material for: Intergenerational transmission of child maltreatment using a multi-informant multi-generation family design
Source: PLoS One. 2020 Mar 12;15(3):e0225839. doi: 10.1371/journal.pone.0225839 (PMC7067458; doi:10.1371/journal.pone.0225839)
Supplement: S8 Table — (DOCX) [file pone.0225839.s010.docx]

**S8 Table. Stepwise multilevel model for abuse and neglect using a multi-informant approach**

|  | Model 1 | Model 2 | Model 3 | Model 4 |
| --- | --- | --- | --- | --- |
| Dependent variable: Perpetrated abuse | | | |  |
| Fixed effects | Coef (se) | Coef (se) | Coef (se) | Coef (se) |
| Intercept | 1.51 (0.07)*** | 0.92 (0.38)* | 1.01 (0.37)** | 1.12 (0.35)** |
| Gender |  | 0.03 (0.11) | 0.03 (0.11) | 0.03 (0.10) |
| Age |  | 0.01 (0.01) | 0.01 (0.01) | 0.01 (0.01) |
| SES |  | -0.06 (0.08) | -0.02 (0.08) | -0.04 (0.07) |
| Reporter convergence |  |  | 0.14 (0.04)** | 0.14 (0.04)*** |
| Mother report |  |  |  | -0.05 (0.13) |
| Father vs. Child |  |  |  | -0.40 (0.13)** |
| Variance components |  |  |  |  |
| Individual level | 0.51 | 0.49 | 0.46 | 0.42 |
| Family level | 0.12 | 0.12 | 0.09 | 0.05 |
| Dependent variable: Perpetrated neglect | | | |  |
| Fixed effects | Coef (se) | Coef (se) | Coef (se) | Coef (se) |
| Intercept | 1.90 (0.07) | 2.14 (0.41)*** | 2.14 (0.41)*** | 2.43 (0.44)* |
| Gender |  | -0.36 (0.12)** | -0.36 (0.12)** | -0.39 (0.12)** |
| Age |  | 0.01 (0.01) | 0.01 (0.01) | 0.01 (0.01) |
| SES |  | 0.03 (0.09) | 0.03 (0.09) | 0.05 (0.10) |
| Reporter convergence |  |  | 0.04 (0.06) | 0.04 (0.06) |
| Child report |  |  |  | 0.14 (0.09) |
| Mother vs. Father |  |  |  | 0.11 (0.20) |
| Variance components |  |  |  |  |
| Individual level | 0.66 | 0.61 | 0.60 | 0.58 |
| Family level | 0.09 | 0.09 | 0.09 | 0.09 |

*Note*. The unstandardized coefficients are represented. * *p* < .05, ** *p* < .01, ****p* < .001
